# Allergic Contact Dermatitis in Psoriasis Patients: Typical, Delayed, and Non-Interacting

**DOI:** 10.1371/journal.pone.0101814

**Published:** 2014-07-24

**Authors:** Maria Quaranta, Stefanie Eyerich, Bettina Knapp, Francesca Nasorri, Claudia Scarponi, Martina Mattii, Natalie Garzorz, Anna T. Harlfinger, Teresa Jaeger, Martine Grosber, Davide Pennino, Martin Mempel, Christina Schnopp, Fabian J. Theis, Cristina Albanesi, Andrea Cavani, Carsten B. Schmidt-Weber, Johannes Ring, Kilian Eyerich

**Affiliations:** 1 ZAUM – Center of Allergy and Environment, Technische Universität and Helmholtz Center Munich, Member of the German Center for Lung Research (DZL), Munich, Germany; 2 Institute of Computational Biology, Helmholtz Center Munich, Neuherberg, Germany; 3 Laboratory of experimental immunology, IDI-IRCCS, Rome, Italy; 4 Department of Dermatology and Allergy, Technische Universität Munich, Munich, Germany; 5 Department of Dermatology, Venereology, and Allergology, University Medicine Goettingen, Goettingen, Germany; 6 Department of Mathematics, Technische Universität Munich, Garching, Germany; Ludwig-Maximilian-University, Germany

## Abstract

Psoriasis is characterized by an apoptosis-resistant and metabolic active epidermis, while a hallmark for allergic contact dermatitis (ACD) is T cell-induced keratinocyte apoptosis. Here, we induced ACD reactions in psoriasis patients sensitized to nickel (n = 14) to investigate underlying mechanisms of psoriasis and ACD simultaneously. All patients developed a clinically and histologically typical dermatitis upon nickel challenge even in close proximity to pre-existing psoriasis plaques. However, the ACD reaction was delayed as compared to non-psoriatic patients, with a maximum intensity after 7 days. Whole genome expression analysis revealed alterations in numerous pathways related to metabolism and proliferation in non-involved skin of psoriasis patients as compared to non-psoriatic individuals, indicating that even in clinically non-involved skin of psoriasis patients molecular events opposing contact dermatitis may occur. Immunohistochemical comparison of ACD reactions as well as *in vitro* secretion analysis of lesional T cells showed a higher Th17 and neutrophilic migration as well as epidermal proliferation in psoriasis, while ACD reactions were dominated by cytotoxic CD8+ T cells and a Th2 signature. Based on these findings, we hypothesized an ACD reaction directly on top of a pre-existing psoriasis plaque might influence the clinical course of psoriasis. We observed a strong clinical inflammation with a mixed psoriasis and eczema phenotype in histology. Surprisingly, the initial psoriasis plaque was unaltered after self-limitation of the ACD reaction. We conclude that sensitized psoriasis patients develop a typical, but delayed ACD reaction which might be relevant for patch test evaluation in clinical practice. Psoriasis and ACD are driven by distinct and independent immune mechanisms.

## Introduction

Psoriasis and allergic contact dermatitis (ACD) are highly prevalent, complex inflammatory skin diseases characterized by a combination of epithelial alterations and deviated T cell immunity [1], [2].

In recent years, substantial progress was made in understanding the pathogenesis of both psoriasis and ACD. However, major scientific and clinical questions remain unresolved. In particular, the question whether psoriasis is primarily based on epithelial or immune alterations is still under debate. Valid arguments are found for both theories: while deletion of the epidermal proteins JunB/activator protein1, IKK2 or STAT3 overexpression are each sufficient to induce a psoriasis-like phenotype in mice [3]–[5], the description of patients with both psoriasis and eczema or ACD in parallel argues for specific local stimuli inducing either psoriasis or eczema [6].

In that context, a promising approach to investigate the pathogenesis of psoriasis and ACD or other dermatitis entities is to compare the molecular changes in lesional skin of both diseases. However, previous attempts to do so were limited by high inter-individual variability caused by different genetic background as well as environmental exposure, skin infections or trauma and eventual systemic or topical treatments [7]–[9]. These obstacles may largely be overcome by the possibility to investigate both diseases in the same patient at the same time. However, a prerequisite for general conclusions from such an analysis is the proof that sensitized psoriasis patients develop typical dermatitis either spontaneously [10] or to iatrogenic epicutaneous challenge with allergens or haptens (ACD), respectively.

Clinically, it should be easy to determine whether psoriasis patients may develop an ACD reaction. Psoriasis is characterized by squamous, well-demarcated red plaques of variable size [11]. In contrast, ACD is mainly characterized by diffuse dryness, erythema, papules, and vesicles followed by scaling [1]. The main histologic features of psoriasis plaques include epidermal hyperplasia with elongated rete ridges and altered keratinocyte cornification, neutrophil microabscesses and a T cellular infiltrate. Histological features of ACD are epidermotropism as well as dermal infiltration of T cells and epidermal spongiosis and apoptosis, reflecting a pathogenic hallmark of ACD: induction of keratinocyte apoptosis by T cells [12]–[15]. This key event opposes the pathogenesis of psoriasis. Here, the typical clinical picture is due to hyper-proliferation, apoptosis resistance and insufficient differentiation of keratinocytes. Thus, it is unclear to which extent psoriasis patients develop typical ACD reactions. This is in line with epidemiological studies reporting a decreased rate in type IV sensitizations in psoriasis patients [16], [17].

The aim of this study was an intra-individual comparison of psoriasis and ACD. We demonstrate that psoriasis patients can develop delayed, but otherwise typical contact dermatitis. Psoriasis and ACD differed in their immune cellular infiltrate and epithelial changes. Induction of ACD reactions directly on top of pre-existing psoriasis plaques did not alter the long-term clinical course of psoriasis, which indicates independent pathogenic mechanisms are the molecular basis of psoriasis and ACD.

## Materials and Methods

### Patients and material sampling

Patients with moderate-to-severe plaque type psoriasis and co-existing type IV sensitizations to nickel (allergic contact dermatitis, n = 14) were included into the study. A detailed clinical characterization of the patients is given in table 1. Exclusion criteria were treatment with immune-efficient medication prior to material sampling (wash-out phase 6 weeks for systemic, 2 weeks for local treatment). Diagnoses were made according to clinical as well as histological criteria as published previously [18]. Clinical evaluation of the patients was quantified using the PASI, and intensity of induced contact dermatitis reactions to nickel was documented according to guidelines as published previously [6]. For comparison of ACD kinetics, non-psoriatic patients admitted to our day care hospital with a positive epicutaneous patch test to nickel (n = 8) were included into the study. For analysis of the molecular changes of clinically non-involved skin, non-psoriatic individuals (patients with lichen planus, n = 3 as well as patients without any skin disease, n = 7) were included. The study was conducted according to the declaration of Helsinki. All patients gave their written consent to participate in the study, and the study was approved by the local ethical committee: Klinikum Rechts der Isar of the Technische Universität Munich (project number 5060/11).

**Table 1:** Clincial characterization of patients enrolled into the study.

Patient	Age	Gender	Psoriasis onset [age]	Psoriasis arthritis	Family history	Grade of ACD reaction
1	46	female	32	no	pos	+++
2	31	female	28	no	pos	++
3	58	female	44	yes	pos	+++
4	56	female	52	no	neg	+++
5	70	female	51	no	neg	+++
6	41	female	26	no	neg	+++
7	46	female	12	no	pos	+++
8	55	female	50	no	neg	+++
9	57	male	43	yes	pos	+++
10	61	female	40	yes	pos	+++
11	52	female	45	no	neg	++
12	42	female	19	no	neg	+++
13	38	female	13	neg	neg	+++
14	48	female	37	neg	neg	++

Six-mm skin punch biopsies were obtained under local anaesthesia from one ACD lesion five days after hapten challenge, one active psoriasis plaque, one ACD reaction directly on top of a pre-existing psoriasis plaque, and clinically non-involved skin of all patients. Biopsies were divided into three parts for routine histological evaluation and isolation of total RNA and primary T cell lines, respectively.

### Isolation of total RNA and cDNA transcription from skin biopsies

Skin tissue specimens were stored in PAXgene Tissue containers (Qiagen) at −20°C. For isolation of RNA, tissue samples were homogenized with a tissue lyzer (Qiagen) followed by the purification of total RNA with the PAXgene Tissue RNA Kit (Qiagen) according to the manufacturer’s protocol. The RNA yield and quality was determined with a Nanodrop ND1000 UV-vis Spectophotomer. cDNA was synthesized from 300 ng total RNA and transcribed using the High Capacity cDNA Reverse Transcript Amplification Kit (Applied Biosystems) according to the manufacturer’s instructions.

### Primer design and Real-time PCR

Primers for real time PCR were designed using the publicly accessible Primer3 software (http://frodo.wi.mit.edu/primer3/). A list of used primers is shown in Table S1.

For amplification of genes of interest by real time PCR, cDNA was amplified using gene-specific primers and the Fast Start SYBR Green Master mix (Roche Applied Science). Reactions were performed in 384-well plates and fluorescence development monitored with a ViiA7 Real Time PCR machine (Applied Biosystems). The expression of transcripts was normalized to expression of 18S ribosomal RNA as housekeeper and fold change, relative to non-involved skin as calibrator, was determined using the ΔΔCt method.

### Whole genome expression arrays

cRNA was synthesized from 25 ng total RNA and transcribed using the High Capacity cDNA Reverse Transcript Amplification Kit (Applied Biosystems) according to the manufacturer’s instructions. Samples were amplified and Cy3 labeled using the Agilent Low Input Quick Amp Labeling Kit (Agilent Technologies) according to the manufacturer’s instructions and hybridized to a SurePrint G3 Human GE 8X60K BeadChip (Agilent Technologies). After washing the hybridized arrays, fluorescence signals were detected by reading the arrays in the microarray scanner system iScan (Agilent Technology). The Agilent Feature Extraction software was used to read and process microarray image files.

Data analysis was performed using limma (Bioconductor, R) [19]. The “normexp” background corrected arrays have been normalized with the method “cyclicloess”. Only probes which are at least 10% brighter than the 95 percentile of the negative control probes were processed further. Control Probes and low expressed probes were filtered out. Differential gene expression was assessed using a two-sample, two-sided Welch’s t-test. To correct for multiple hypothesis testing the Benjamini and Hochberg approach (FDR) has been used. Fold-changes (FC) were computed using mean values and expressed as logarithm base 2. Significant differentially expressed genes were defined with a significance threshold of corrected p-values <0.05 and an absolute log FC cutoff of 1. The “weight01” algorithm of the topGO package was used to determine significantly enriched GO terms.

### Immunohistochemistry

Skin samples were fixed in 10% formalin and embedded in paraffin. 5-µm sections were de-waxed and rehydrated. After quenching endogenous peroxidase, achieving antigen retrieval, and blocking nonspecific binding sites, sections were incubated with monoclonal antibodies against CD15 (BD-Pharmingen, Franklin Lakes, NJ, 1∶100 dilution), CD3 (BD-Pharmingen, 1∶20 dilution), BDCA-2 (Miltenyi Biotec, Bergisch Gladbach, Germany), Ki67 (Dako, Carpinteria, CA, mib1 clone, 1∶50 dilution), IL-17 (R&D Systems, Minneapolis, MN, 1∶40) and perforine (Leica Biosystems, Milan, Italy, 5B10 clone, 1∶20). Secondary biotinylated mAbs and staining kits were obtained from Vector Laboratories. Stainings were developed using 3-amino-9-ethylcarbazole (Vector Laboratories, Burlinagame, CA, USA). Slides were counterstained with hematoxylin. As a negative control, primary antibodies were omitted or replaced with an irrelevant isotype-matched monoclonal antibody. Figures depict one experiment that is representative of all the patients investigated (n = 10). Positive cells were counted in two visual fields per condition by two independent investigators in a blinded manner.

### Blood flow analysis and clinical evaluation

To investigate whether ACD reactions might influence pre-existing psoriasis plaques, blood flow analysis of the resulting skin lesions was performed using laser Doppler imaging (Moor instruments) as previously published [20]. Additionally, psoriasis lesions were scored before and 21 days after nickel challenge according to local PASI criteria: erythema, squamation, and infiltration each ranging from 0 =  none to 3 =  severe. All values were added to calculate the total local PASI score.

### Isolation of skin-infiltrating T cells

Skin biopsies were minced with a scalpel and placed in culture RPMI medium supplemented with 2 mM glutamine, 1 mM sodium pyruvate, 1% nonessential amino acids, 1% penicillin/streptomycin (all Invitrogen; RPMI complete), and 10% human serum (Sigma-Aldrich) 60 U/ml IL-2. After 2–5 d, T cells emigrated from tissue samples according to the IL-2 gradient and were further expanded by stimulation with aCD3/aCD28 antibodies (BD Biosciences).

### Bioplex analysis

T cells isolated from skin biopsies were stimulated in RPMI complete and the presence or absence of aCD3/aCD28 antibodies for 48 hours. Cell-free supernatant was obtained and stored at −80°C until further analysis. Bioplex analysis was performed using the Bio-Plex Pro Human Cytokine 27-Plex Assay (Bio Rad) according to the manufacturer’s protocol. Quantification of protein content was determined with the Bio-Plex 200 System (Bio Rad).

### Statistical analysis

Statistical analysis was performed using the R software (http://www.r-project.org). A two-sample, two sided paired Welch’s *t*-test was used to compare released factors and cellular infiltrate in psoriasis and ACD reactions. We corrected for multiple hypothesis testing using the Benjamini and Hochberg method (FDR). A corrected p-value <0.05 was considered significant.

## Results

### Contact dermatitis is delayed in psoriasis patients

**Figure 1:**
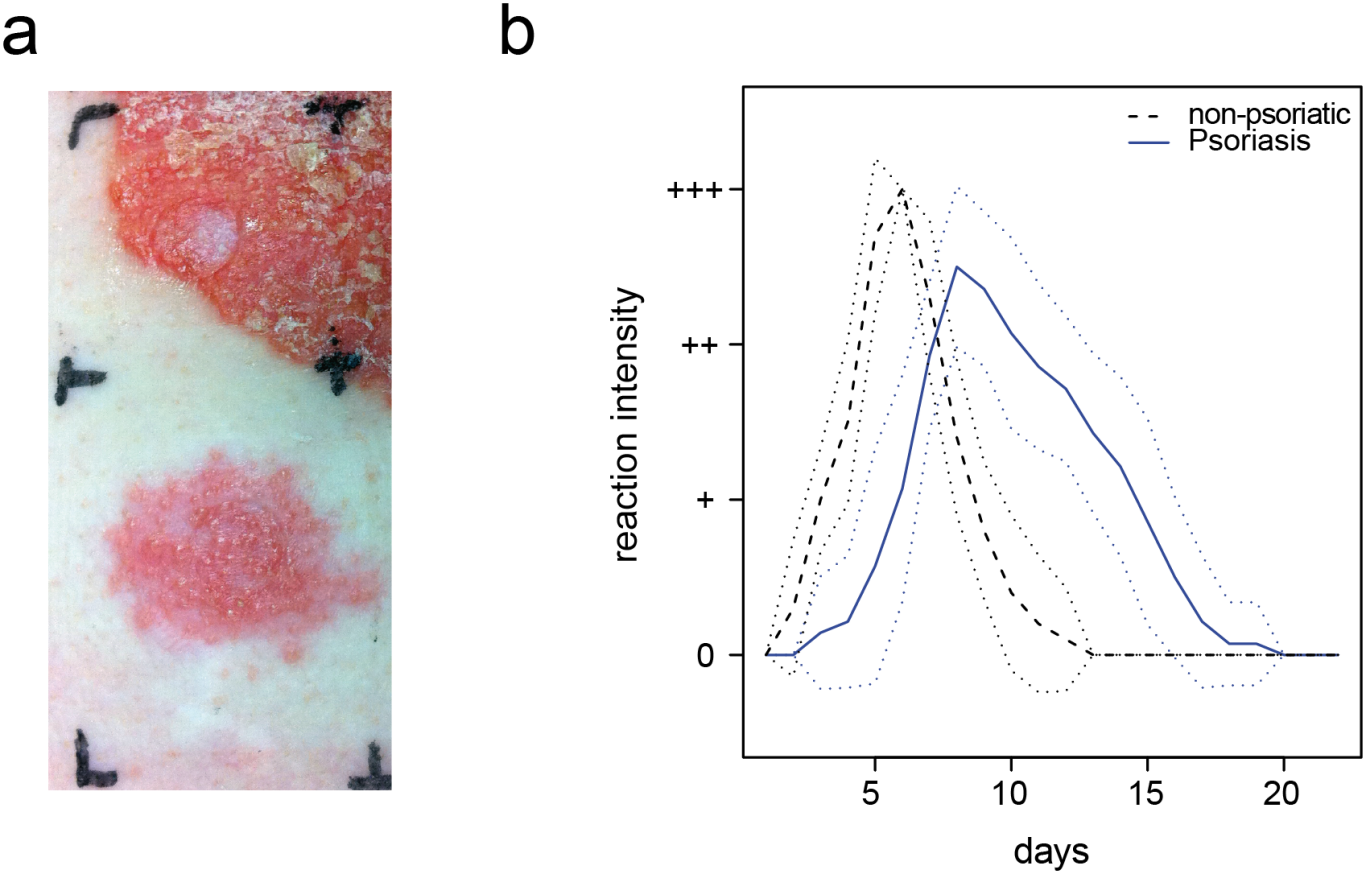
Induction of allergic contact dermatitis in psoriasis patients. **A** Dermatitis 5 days after application of a nickel patch test in close proximity to a psoriasis plaque; **B** Comparison of reaction severity and time course of nickel patch test reactions to nickel in sensitized psoriasis patients (n = 14, mean is shown in blue line) and non-psoriatic individuals (n = 10, mean is shown in black dashed lines). Standard deviation is given by dotted lines.

As a first step to investigate whether sensitized psoriasis patients develop a typical ACD reaction, we performed nickel patch tests in patients with plaque-type psoriasis and a known hypersensitivity reaction to nickel (n = 14). Epicutaneous application of nickel induced dermatitis in all patients, as characterized by diffuse erythema, papules, and vesicles followed by scaling even in close proximity to pre-existing psoriasis lesions with the typical clinical presentation of squamous, well-demarcated red plaques of variable size (Fig. 1A).

As compared to non-psoriatic nickel sensitized individuals (n = 10), the psoriasis nickel-sensitized patients showed a delayed time course of the ACD reaction (Fig. 1B). While non-psoriatic patients usually developed a positive patch test reaction after 48–72 hours, patch tests were frequently still negative at that time point in the case of psoriasis patients. In line with that observation, the maximum intensity of the clinical reaction was reached after 3–5 days in the non-psoriatic group, but after 7 days in the psoriasis group. ACD reactions in the psoriasis patients showed also a slower resolution as compared to non-psoriatic patients (Fig. 1B).

### Clinically non-involved skin of psoriasis patients shows altered metabolism and proliferation pathways

**Figure 2:**
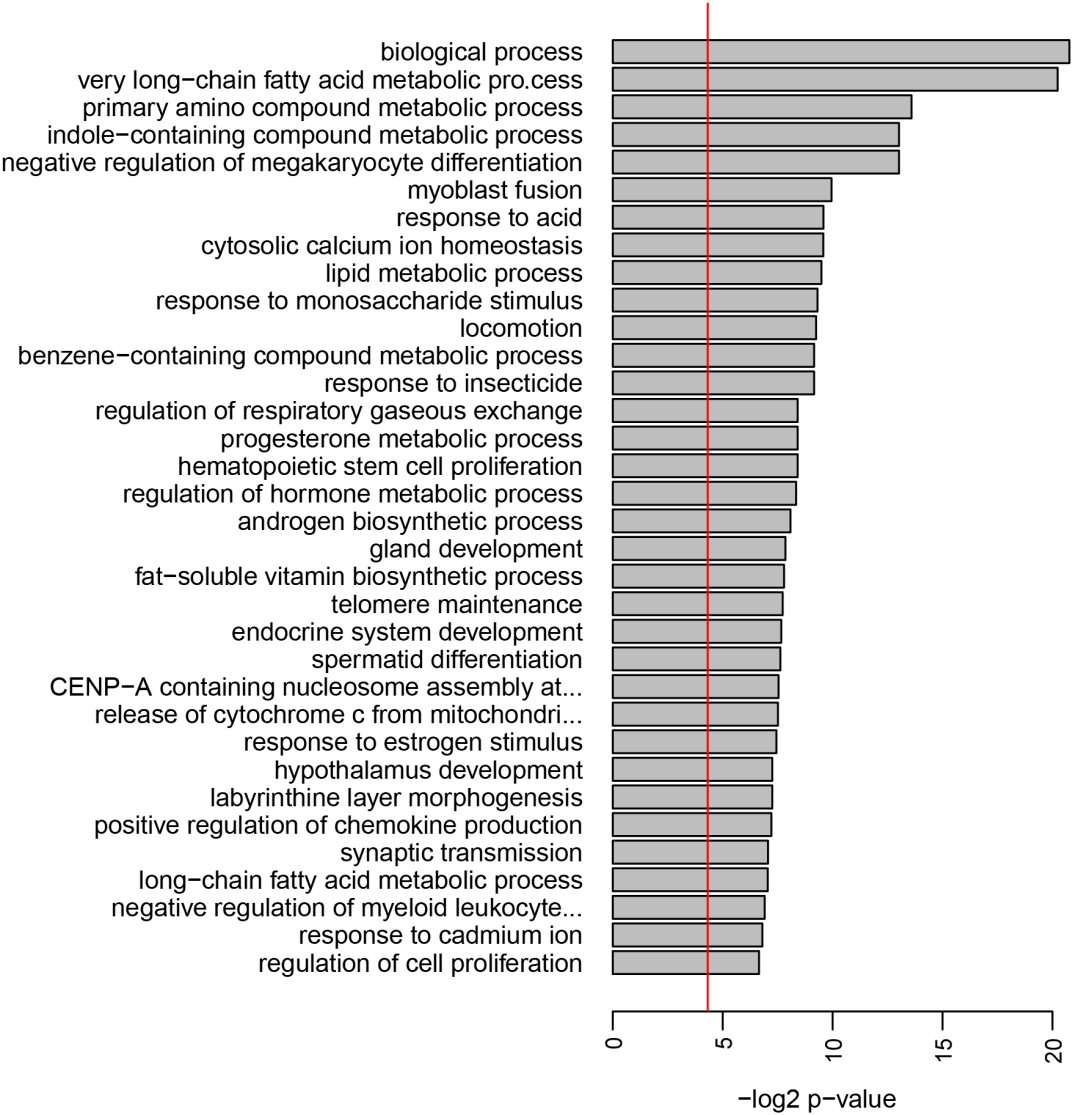
Clinically non-involved skin of psoriasis patients is altered regarding metabolism and proliferation. Signaling pathway analysis of clinically non-involved skin of psoriasis patients (n = 8) versus healthy skin of non-psoriatic individuals (n = 10). Shown are the most significant hits for the Gene Ontology term “biological process”. The bar size indicates the level of significance for each pathway (negative log2 p-value), the vertical line shows the 0.05 significance level.

To understand the molecular basis of the delayed ACD time course in psoriasis patients, whole genome expression analysis of clinically non-involved skin of psoriasis patients as compared to non-psoriatic individuals was performed. Mean expression of 531 genes (1293 probes) was significantly different in the psoriasis group. A GO term pathway analysis showed that numerous pathways related to metabolism of lipids, amino acids, saccharides, and hormones as well as proliferation and differentiation pathways such as telomere maintenance, stem cell proliferation, and regulation of cell proliferation, were altered in psoriasis patients even in clinically non-involved skin (Fig. 2).

### Psoriasis plaques show a Th17 profile while ACD reactions are dominated by cytotoxic T cell responses *in situ*

**Figure 3:**
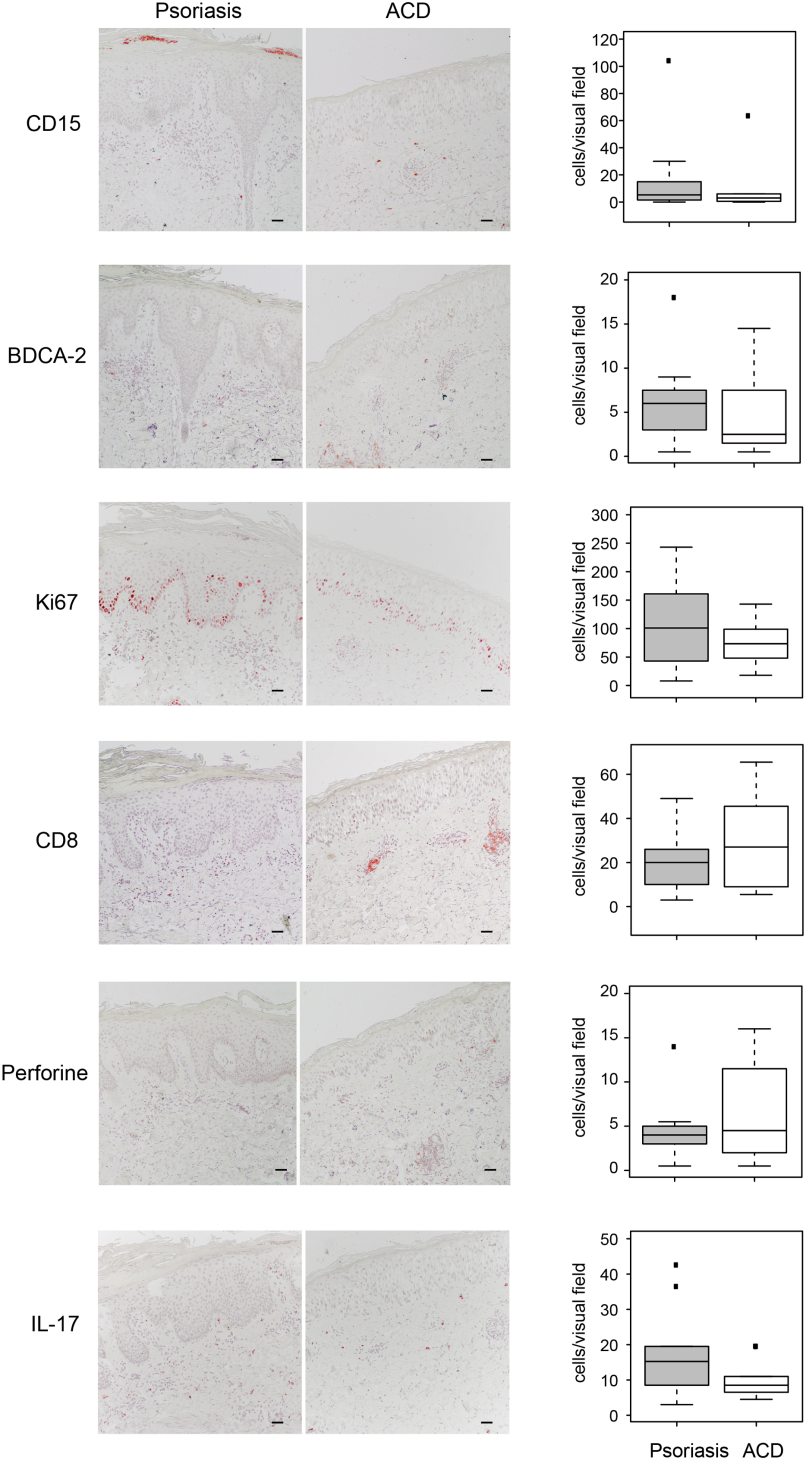
Immunohistochemical comparison of psoriasis and ACD reactions in the same patient. Representative immunohistochemical stainings of psoriasis and ACD biopsy sections obtained from the same patient (right panel) and quantification of positive cells per visual field of all included patients (n = 10) (boxplots in left panel).

To further investigate the pathogenesis of psoriasis and ACD, we performed immunohistochemical studies on skin biopsies of acute psoriasis plaques and positive nickel patch test reactions. In psoriasis plaques, non-significant trends of a higher infiltration of CD15+ neutrophil granulocytes (17.1 *versus* 8.9 positive cells per visual field, p = 0.5), BDCA-2+ plasmacytoid dendritic cells (6.3 *versus* 4.5, p = 0.3), and IL-17+ T cells (17.8 *versus* 10.0, p = 0.3) as compared to ACD reactions was observed. Furthermore, the proliferation marker Ki67 was expressed higher in psoriasis plaques than in ACD lesions (108.6 *versus* 73.6, p = 0,3). Positivity for Ki67 was observed especially in basal layers of the epidermal compartment, but was also observed in higher epidermal layers in the case of psoriasis (Fig. 2). In contrast, a higher number of cytotoxic CD8+ T cells (29.2 *versus* 20.7, p = 0,46) and perforine positive cells (6.5 *versus* 4.6, p = 0,46) was observed in ACD reactions as compared to psoriasis plaques (Fig. 3).

### T cell secretion analysis reveals differences between psoriasis and ACD regarding the infiltrating T cell compartment

**Figure 4:**
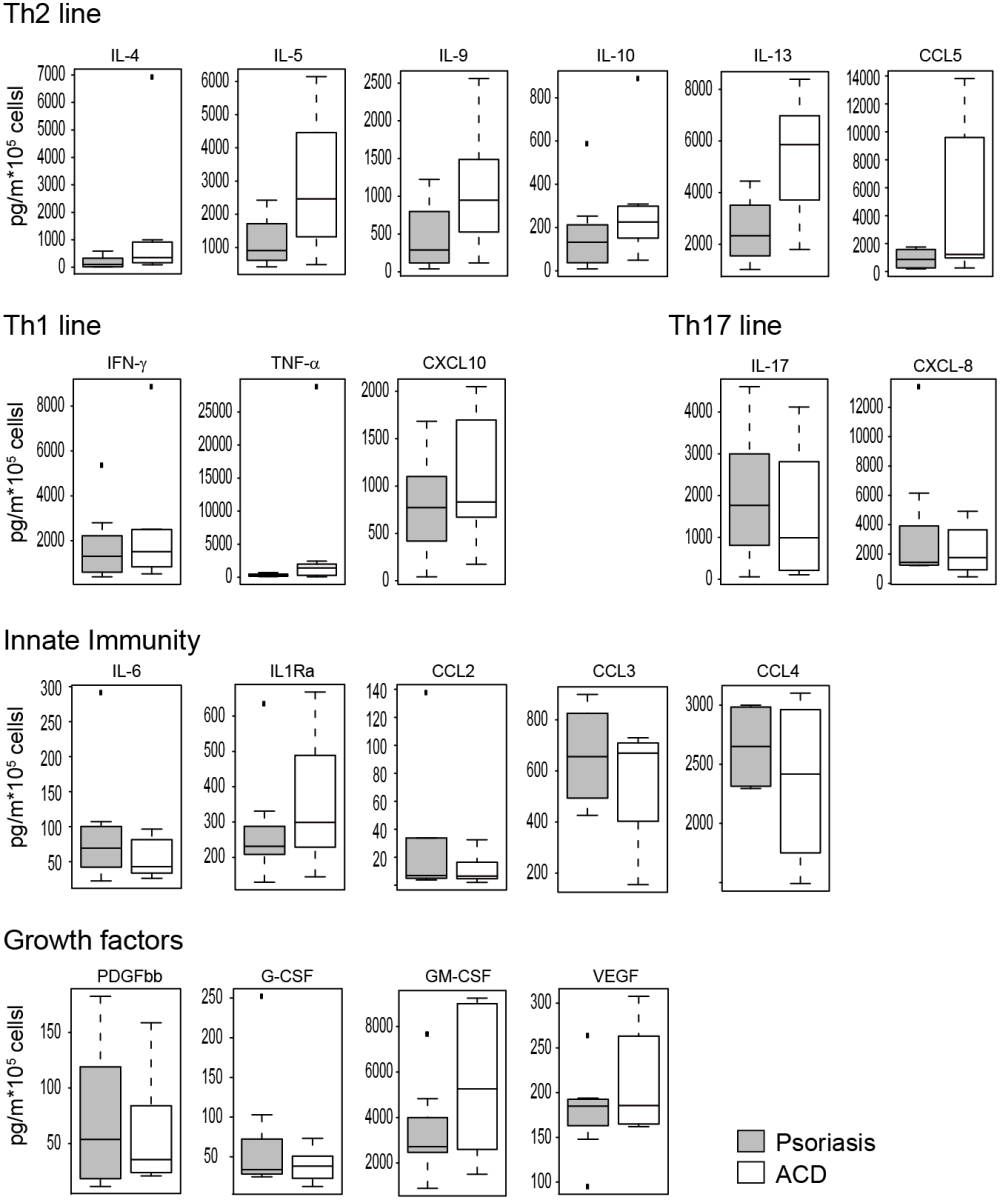
Bioplex analysis of skin infiltrating T cells. T cells infiltrating either the psoriatic plaque or the induced ACD lesion were stimulated with aCD3/aCD28 antibodies and cell-free supernatant was used for Bioplex analysis with 27 different analytes. Detected proteins were grouped according to association with the Th2 line (**A**), Th1 line (**B**), Th17 line (**C**), innate immunity (**D**) and growth factors (**E**). Shown are boxplots from five patients.

Lesional T cells were isolated from psoriasis plaques and positive ACD reactions simultaneously and analyzed *in vitro* (Fig. 4). Upon anti-CD3/anti-CD28 stimulation, psoriasis infiltrating T cells released more IL-17 (2.0 *versus* 1.5 ng/ml, p = 0.85) than ACD derived T cells. In contrast, the Th2 cytokines IL-4 (1.2 *versus* 0.2 ng/ml, p = 0.18), IL-5 (2.9 *versus* 1.2 ng/ml, p = 0.67), IL-9 (1.1 *versus* 0.5 ng/ml, p = 0.23), IL-10 (0.3 *versus* 0.2 ng/ml, p = 0.18), and IL-13 (5.3 *versus* 2.5 ng/ml, p = 0.96) showed a consistent trend of higher secretion in ACD as compared to psoriasis T cells. No marked difference was observed regarding the Th1 cytokine IFN-γ (2.4 *versus* 1.8 ng/ml, p = 0.38). IL-6 was secreted at low levels with slightly more in psoriasis plaque T cells (0.09 *versus* 0.05 ng/ml, p = 0.18), while the soluble IL-1 receptor α (0.36 in ACD *versus* 0.28 ng/ml in psoriasis, p = 0.18) and TNF-α (4.5 *versus* 0.3 ng/ml, p = 0.18) were secreted in higher amounts by ACD T cells. The Th17 chemokine CXCL8 was secreted to a higher amount by psoriasis-derived T cells (3.5 *versus* 2.3 ng/ml, p = 0.72), while the Th2-associated chemokine CCL-5 was released more by ACD T cells (4.5 *versus* 0.9 ng/ml, p = 0.53). No marked differences were observed regarding secretion of the Th1 chemokine CXCL10 as well as of CCL2, CCL3, and CCL4 (Fig. 4). Lesional T cells also released growth factors. PDGF-bb and G-CSF were expressed at very low levels, GM-CSF (5.5 *versus* 3.4 ng/ml, p = 0.67) and VEGF (0.21 *versus* 0.18 ng/ml, p = 0.18) were slightly higher released by ACD T cells.

### Induction of an ACD reaction on a pre-existing psoriasis plaque results in a mixed phenotype of both diseases

**Figure 5:**
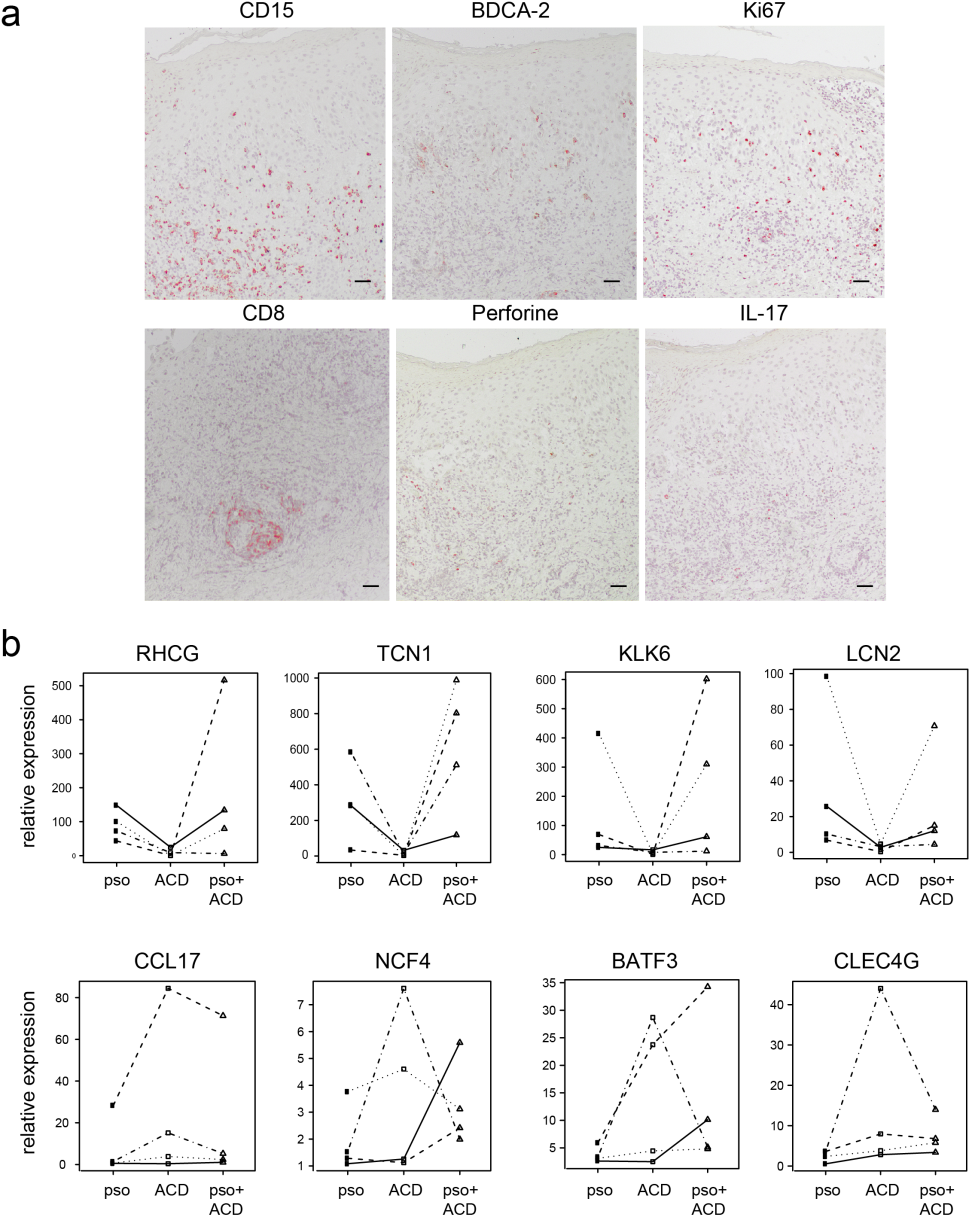
Characterization of ACD on top of psoriasis plaques. ACD was induced by applying a nickel patch test on top of a pre-existing psoriasis plaque. **A** Immunohistochemical evaluation of hallmarks of ACD and psoriasis. **B** Real time PCR analysis of genes specific for psoriasis (upper panel) and ACD (lower panel). Non-involved skin served as control and data is shown as fold induction (2^−ΔΔCT^). Each line indicates samples coming from the same patient.

To investigate a possible direct interaction of psoriasis and ACD, we challenged nickel-sensitized psoriasis patients with nickel on top of a pre-existing, active psoriasis plaque in 11 patients. Resulting inflammatory reactions were analyzed histologically. Histologic hallmarks of both diseases were observed, namely psoriasiform acanthosis with neutrophilic micro-abscesses and dilated capillaries in the papillary dermis for psoriasis and marked spongiosis, epidermotropism and keratinocyte apoptosis defining dermatitis (Fig. 5A).

In addition, immunohistochemical investigation demonstrated that markers typical for both psoriasis and ACD, such as CD15, IL-17, and BDCA-2, and the cytotoxic marker CD8, respectively, were simultaneously present in the mixed psoriasis/ACD lesion (Fig. 5A). The expression of immune cells was spread in both dermis and epidermis and mostly distributed at the site of epidermal spongiosis and micro-vesiculation. Thus, the distribution of immune cells reflected the course of an acute inflammatory reaction (Fig. 5A).

### Disease-specific psoriasis and ACD genes are constant in the mixed phenotype reaction

In order to assess whether the psoriasis and ACD reactions could influence each other, previously identified transcripts specific for psoriasis or ACD [21] were quantified in biopsies from psoriasis, ACD, and psoriasis/ACD skin lesions of the same patient as compared to non-involved skin. Namely, *RHCG, TCN1, KLK6,* and *LCN2* were chosen for psoriasis and *CCL17, NCF4, BATF3,* and *CLEC4G* as ACD-specific genes. Results revealed a strong up-regulation of the psoriasis-specific genes in psoriasis plaques, but not in ACD reactions. This was true for all markers investigated: *RHCG* (91 *versus* 9-fold up-regulation), *TCN1* (285 *versus* 8-fold up-regulation), *KLK6* 50 *versus* 5-fold up-regulation), and *LCN2* (18 *versus* 3-fold up-regulation) in psoriasis and *CCL17* (1-fold *versus* 10-fold up-regulation), *NCF4* (1.4-fold *versus* 3-fold up-regulation), *BATF3* (3-fold *versus* 14-fold up-regulation), and *CLEC4G* (3-fold *versus* 6-fold up-regulation) in ACD. In the mixed psoriasis/ACD lesion, up-regulation of all markers was similar to the up-regulation in the corresponding single disease (Fig. 5B). Thus, psoriasis-specific genes are not markedly influenced by the simultaneous induction of an ACD reaction and *vice versa*.

### The clinical course of psoriasis is not altered by ACD

**Figure 6:**
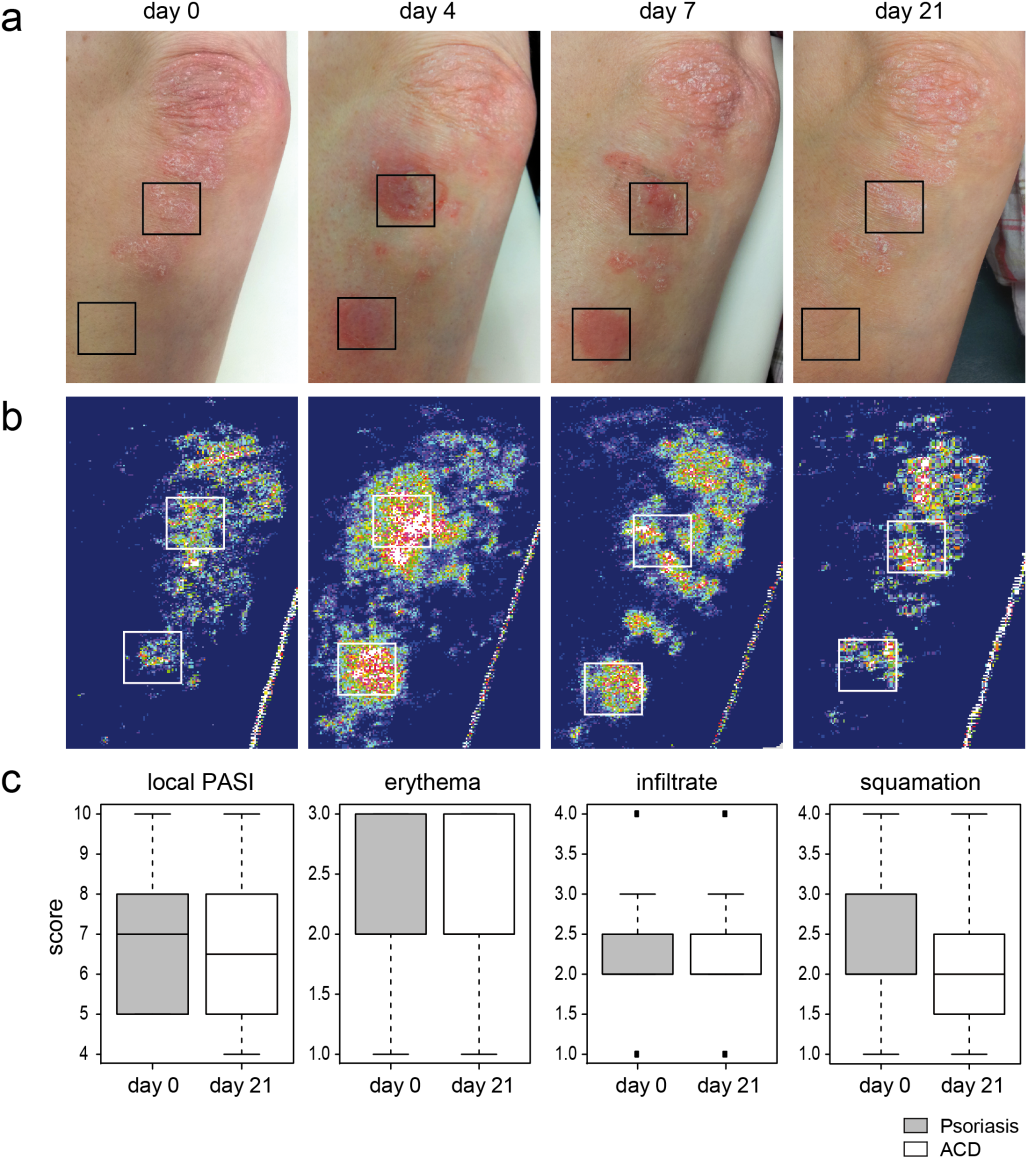
ACD and psoriasis are independent, stimulus-driven immune reactions. Nickel patch tests were used to induce ACD lesions in close proximity to or on top of a psoriasis plaque (n = 11). **A** Representative clinical course of psoriasis, ACD, and psoriasis/ACD mixed reaction in one patient. **B** Subjective clinical evaluation was confirmed investigating the local blood flow as indicator for severity of inflammation via laser doppler imaging at day 0, day 4, day 7 and day 21 (**B**). **C** Changes in local PASI, erythema, infiltrate and squamation were determined for the nickel treated psoriasis plaque before and 21 days after nickel challenge (n = 11) and presented as boxplots.

Since a specific and independent molecular signature was consistent for both psoriasis and ACD even upon ACD challenge on top of a psoriasis plaque, we sought to investigate whether the natural clinical course of these two conditions would be influenced by each other. Clinically, epicutaneous application of nickel on psoriasis plaques induced a strong local inflammatory reaction already after 48 hours in sensitized individuals. This inflammatory reaction was more severe than the nickel patch test reaction on previously non-involved skin, but it cleared following the natural course of an ACD reaction within two weeks. After 3–4 weeks, ACD reactions had resolved completely, but the initial psoriasis plaque remained largely unaltered (Fig. 6A). The subjective clinical investigation was confirmed by a laser blood flow analysis of the cutaneous reactions (Fig. 6B). This blood flow analysis confirmed that despite the strong temporary inflammation, the clinical course of psoriasis was not influenced by the local induction of an ACD reaction. This was confirmed by the degree of the local psoriasis area-and severity index (PASI), erythema, squamation and infiltration. All these parameter were similar, even if slightly reduced, in the psoriasis plaque after three weeks as compared to the plaque before nickel challenge (n = 11; Fig. 6C).

## Discussion

Patients that suffer from psoriasis and ACD simultaneously represent an interesting model to investigate common and distinct molecular mechanisms underlying those two disease patterns.

Dermatitis reactions can be elicited by epicutaneous challenge of common allergens or haptens in sensitized psoriasis patients (allergic contact dermatitis, ACD). This iatrogenic induction offers the possibility to control location and kinetics of the dermatitis response and thus standardizes the experimental setup. However, a prerequisite is that sensitized psoriasis patients develop an ACD reaction similar to non-psoriatic patients. This is doubtful, since hallmarks of psoriasis are antagonistic to ACD. Namely, keratinocytes of psoriasis patients show a higher proliferation and are less susceptible to apoptosis both *in vivo* and *in vitro* [22], [23]. In contrast, ACD is based on early loss of keratinocyte adherence and induction of apoptosis [14], [24]. In fact, when we compared ACD reactions to nickel of psoriatic and non-psoriatic individuals, we observed a delayed time course in psoriasis patients. This is likely due to the observed sub-clinical alterations in clinically non-involved skin of psoriasis patients, where numerous genes related to metabolism and proliferation are altered as compared to healthy skin of non-psoriatic individuals. Additionally, migration of antigen-presenting cells into the local lymph node is delayed in psoriasis patients [25]. These phenomena may also explain why some studies describe an inverse relationship of psoriasis and contact dermatitis reactions [16], [17]. The delayed time course observed in this study implies that epicutaneous patch tests should be additionally evaluated after seven days in psoriasis patients in daily clinical routine.

Beyond the delayed onset, ACD reactions in psoriasis patients were similar to non-psoriatic individuals regarding all clinical and histological parameters even in close proximity to pre-existing psoriasis plaques. This strengthens the concept that not only ACD, but also psoriasis is driven primarily by immune responses to specific stimuli [6]. Recent evidence argue for epidermal antigens such as desmoglein 3 as potential trigger of a psoriatic T cell response [26]. While in line with the literature, in nickel-induced ACD a Th2 and a cytotoxic immune response mediated by CD8+ T cells was more frequent than in psoriasis [18], psoriasis lesions were infiltrated by a higher number of IL-17+ immune cells [27]. A trigger of psoriasis may be a complex of antimicrobial peptides and self-DNA that is released after cellular damage and consecutively activates plasmacytoid dendritic cells to drive a Th17 dominated immune response [28]. This observation fits with the fact that psoriasis shares several aspects of a wound healing reaction [29], [30]. Interestingly, numbers of plasmacytoid dendritic cells were comparable in psoriasis and ACD in our patients, but none developed a psoriatic lesion at the site of hapten challenge. This fact highlights that the specific process of epidermal apoptosis observed in ACD is not sufficient to initiate the pathologic cascade described in psoriasis plaques.

The model described here can further be used for interventional studies. Since the common psoriasis therapy dithranol clears psoriasis lesions by an inflammatory reaction of unknown basis [31], we thought whether induction of an ACD reaction right on top of a pre-existing psoriasis plaque might influence the clinical course of the psoriasis plaque. Our hypothesis was further strengthened by our observation that the ACD reaction was associated with an influx of Th2 cells, since IL-4 was shown to be an efficient psoriasis therapy [32]. As expected, nickel induced a strong inflammatory reaction on top of the psoriasis plaque. In contrast to ACD reactions on non-involved skin, kinetics of ACD on top of psoriasis was not delayed, which indicates common innate immune mechanisms of ACD and psoriasis. Histological analysis of the resulting lesions revealed a mixed phenotype of psoriasis and ACD, with acanthosis and neutrophilic micro-abscesses as well as spongiosis and epidermal apoptosis. Surprisingly, within three weeks the self-limited ACD reaction cleared and left a psoriasis plaque closely resembling the one prior to ACD induction. This means the specific inflammatory reaction induced by nickel acting via Toll like receptor 4, activation of the inflammasome, and direct induction of keratinocyte apoptosis [33]–[35] results in epidermal damage, but not in elimination of the psoriasis trigger. It further means that dithranol acts beyond induction of an unspecific inflammation, maybe via development of free radicals [36] or induction of keratinocyte apoptosis via mitochondrial action [37]. Finally, this observation implies that the factors leading to limitation of the ACD such as hapten-specific regulatory T cell responses are either ACD-specific or not strong enough to limit a psoriasis plaque formation.

In summary, we demonstrate that nickel sensitized psoriasis patients develop a delayed, but otherwise typical ACD reaction to nickel even in close proximity to pre-existing psoriasis plaques. Intra-individual comparison of the pathogenesis of psoriasis and ACD reveals a dominated Th17 response in psoriasis and a stronger Th2 and cytotoxic immune response in ACD. Psoriasis and ACD are distinct immune-driven reactions that do not influence each other.

## Supporting Information

Table S1 Sequences of the primers used for real-time PCR analysis.
(DOCX)
