# Supplementary material for: Allergic Contact Dermatitis in Psoriasis Patients: Typical, Delayed, and Non-Interacting
Source: PLoS One. 2014 Jul 24;9(7):e101814. doi: 10.1371/journal.pone.0101814 (PMC4109932; doi:10.1371/journal.pone.0101814)
Supplement: Table S1 — Sequences of the primers used for real-time PCR analysis. (DOCX) [file pone.0101814.s001.docx]

**Supplementary materials**

**Table S1**

| **Target** | **Sequences** |
| --- | --- |
| *NCF4* | 5’- TCGTCATCGAGGTGAAGACA -3`  5’- TCATGTAGGCGTTGAGGGCA -3` |
| *BATF3* | 5’- ATCTTCTCGTGCTCCTTCAG-3`  5’- AGGACTGCGTGGAGATCTATATCA -3` |
| *LCN2* | 5’- TGCCACCATCTATGAGCTGA -3`  5’- AGTCCTGATCCAGTAGTCAC -3` |
| *CCL17* | 5’- CACTGAAGATGCTGGCCCTG -3`  5’- GAATGGCTCCCTTGAAGTACTC -3` |
| *CLEC4G* | 5’- GAAGCAGACGGCGGCGCTGGGT -3`  5’- TCTCCTGCTCCATCAGCTTC -3` |
| *TCN1* | 5’- GTCAACCACTTCACTCCTG -3`  5’- AGGACAGCCATTGCACCAGTA -3` |
| *RHCG* | 5’- CAGCTGCTCATCATGACTTTCTTCC -3`  5’- CTGTCTCTCCTTGCTCTGCTCTAGGT-3` |
| *KLK6* | 5’- ACTATGATGCCGCCAGCCAT -3`  5’- ACCAGGTGGATGTATGCACACTG -3` |

***Table S1***. Sequences of the primers used for real-time PCR analysis.
